# Responding to the Psychological Needs of Health Workers During Pandemic: Ten Lessons From Humanitarian Work

**DOI:** 10.1017/dmp.2020.356

**Published:** 2020-09-10

**Authors:** Elena Cherepanov

**Affiliations:** Cambridge College, Graduate School of Psychology and Counseling, Boston, Massachusetts

**Keywords:** frontline health workers, complex emergency, COVID-19 response, fantasy, peer support, self-awareness, suicide prevention

## Abstract

When a complex emergency (CE) overwhelms infrastructure, the ability of health-care providers to work efficiently under duress saves lives. The author uses her experience of providing mental health supports to humanitarian aid workers and the pieces of training conducted for internal medicine practitioners to offer guidance on how to manage severe job-related stresses during the response to the coronavirus disease 2019 (COVID-19) pandemic. This work reminds responders about their professional mission and purpose, but its extreme physical and mental demands can take a toll on their well-being and health. In CEs, the sheer volume of work and the emotional over-engagement tend to produce toxic fantasies (eg, rescuer or helper fantasies), acting upon which threatens integrity of care and increases risks for both patients and providers. Accumulated fatigue and exposure to mass suffering and mortality can change the perceived value of life and increase reckless, risk-taking, and suicidal behaviors. Introducing a self-awareness framework prioritizes the awareness of the available choices and making situation-appropriate and informed decisions about balancing one’s own and others’ needs. The COVID-19 response has demonstrated that fostering peer supports, changing organizational culture, addressing self-awareness within a training and supervisory context, and strengthening supports for managers are important parts of disaster preparedness. It also revealed that more research is needed to better understand and meet the special psychological needs of health-care responders.

“You psychologists don’t understand. We don’t talk about feelings, and we cannot relax or meditate, or we would not be able to do our work”—A paramedic

During a complex emergency (CE), when infrastructure is overwhelmed and resources are limited, health-care workers play a key role in mitigating its impact. This line of work creates unprecedented professional and personal challenges as well as unprecedented solutions^1^; understanding this experience can offer practical guidance for managing the psychological needs of the frontline health-care workers.

The response to coronavirus disease 2019 (COVID-19) has revealed how ill-prepared health-care systems were to support these responders but also offers an opportunity to evaluate what needs to be done differently to prepare for future emergencies. Despite ongoing criticism for not doing enough to support the aid workers,^2^ humanitarian work has accumulated a great deal of experience that is relevant to work during CEs and can offer valuable lessons on what to expect and how to manage the extreme work-related stresses. As a global mental health specialist, I responded to CEs in Chernobyl, Chechnya, Spitak earthquake, Kosovo, Ukraine, Liberia, and Abkhazia where I provided direct services to the affected communities, support systems development, trainings for the health providers, and psychological support to the humanitarian aid workers. I have drawn upon this experience and the materials from the series of trainings conducted for internal medicine practitioners during the COVID-19 response to compile 10 lessons that carry significance to shaping response to the pandemic (Table 1). Further research is needed to better understand the unique psychological needs of the health providers during the pandemic, and to develop the effective evidence-based systems of interventions and supports.

**TABLE 1:** Ten Lessons from Humanitarian Response to Complex Emergency

1. It Is Impossible to Prepare for CEs, but Their Impact Can Be Mitigated: A Perfect Storm	The impact of CE is complicated by the disruption in the fabric of social life, and the health providers are being a part of the affected community
2. CEs Remind Health Professionals About Their Mission and Purpose	When responding to CE, providers get the opportunity to directly contribute to transformative change in the society and health-care system
3. Frontline Workers Have Different Psychological Needs in Different Phases of the Emergency	Responders have different psychological needs during the Mobilization, Resistance, and Reentry phases of the pandemic response
4. Some Stressors Are Worse Than Others: “Don’t Call Us Heroes”	Uncertainty about the future, a sense of helplessness, worries about family’s safety, and problematic social supports can leave responders emotionally drained and physically exhausted
5. Peer Support	Peer-to-peer support is the support of choice during CE. Its objective is to make peers feel noticed, understood, supported, and validated. It does not substitute professional assistance in extreme cases
6. Anger Is Everywhere and It Masks Other Feelings	Recognizing the underlying feelings of helplessness, sadness, and fear and expressing them for what they are can help to manage anger
7. Recognizing and Addressing Fantasies	Acting out the Rescuer, Supermen or Helper’s fantasies threatens the integrity of care, carries the potential for boundary violation and can put the patients and responders at risk
8. Self-Awareness Offers Choices	The awareness of one’s limits and needs empowers providers to make informed decisions about safety and self-care that are most appropriate for the situation and the professional role
9. Suicide Risk Among Frontline Workers Is Real!	The exposure to massive suffering and mortality may change the perceived meaning and value of life, resulting in recklessness, taking unnecessary risks, and suicidal ideations
10. It Is Easier to Adapt to Emergency Than to Reentry	Various studies of emergency responders have demonstrated that reentry, if not addressed, has been associated with increased risks for substance abuse, depression, family breakup, and suicidal behavior

## IT IS IMPOSSIBLE TO PREPARE FOR CES, BUT THEIR IMPACT CAN BE MITIGATED

A CE is a type of disaster event that is caused by and results in a complicated set of social, medical, and often political circumstances, usually leading to great human suffering and death, and requiring a broad and integrated response.^3^ Often, CEs occur in the context of war or epidemic; other examples include the Chernobyl disaster in 1986, the cholera epidemic in Haiti in 2010, Fukushima disaster in 2011, and the 2014-2016 Ebola epidemic in West Africa.

Although the scale of the system failure in low-resource settings that already struggle with lack of resources cannot be compared with the robust health-care systems in developed countries, conceptualizing the COVID-19 pandemic as a form of CE allows for placing the challenges of the response into a broader context. Various CEs share similar characteristics^4^:

- It is impossible to be fully prepared for a CE.
- Its impact on the community and the responders is systemic and multilevel.
- It’s impact profoundly disrupts the fabric of social life.
- It overwhelms human resources, infrastructure, and logistic capability.
- Its responders can be the members of the same community and are subjected to the same ailments as their patients.

I further argue that, in the health-care system, CEs typically generate internal conflicts between

- the extensive needs and limited human and logistical resources;
- the emergency mode of operations and a heavily regulated health-care system;
- the needs for planning and preparedness and the demands of a rapidly changing situation; and
- having to provide patient care and not being able to ensure one’s own and family’s safety.

## CEs REMIND HEALTH PROFESSIONALS ABOUT THEIR MISSION AND PURPOSE

Despite of all the hardship and confusion, work in a CE potentially can become a life-changing and rewarding experience. It underscores the value of camaraderie and strengthens peer bonding. For the mission-driven helping professions, it serves as a reminder of why we chose to do this work in the first place. CEs remind us of the fragility of what we took for granted, and teach us to appreciate what we have.

They also expand the understanding of the professional role to include fighting stigma, advocacy, and temoignage. Fighting stigma becomes an important component of community education because epidemics are often associated with stigmatization of the survivors.^5,6^ Temoignage (or bearing witness) means that witnessing inequity and injustice in accessing health care adds more power to the advocacy. In this way, providers get the opportunity to use their experience to directly contribute to transformative change in the health-care system.

## FRONTLINE WORKERS HAVE DIFFERENT PSYCHOLOGICAL NEEDS IN DIFFERENT PHASES OF THE EMERGENCY

The experience and psychological needs of the responders can be different at different stages of the disaster response, which are loosely based on Selye’s stress response classification^7^ and is categorized as mobilization, resistance, and reentry.^2^

### Mobilization

*“It is pretty intense. I cannot prepare or plan for anything: every new day changes everything and I come to a completely different workplace. I cannot get it out of my mind. I think about it all the time, even when I sleep, eat, or make love” (personal communication).*

During the initial response, frontline workers push the limits of their endurance and are often surprised by how much they are able to accomplish. This state of extreme mobilization and concentration I call *a functional dissociation* and define as an almost dissociative state of mind that allows the individual to remain functional and focused through extreme stress. This state of mind allows responders to remain efficient when working “crazy hours,” but it is not sustainable in the long term. Psychological supports at this phase are aimed at minimizing its long-term impact on health.

### Resistance

*“All my days are clumped together: I don’t know how many days passed and I don’t care. I don’t even know what day it is—they are all the same, and my days off feel strange” (personal communication).*

At this phase, the emergency becomes a routine. The frontline workers realize that they can continue working longer than they thought they could. During this phase, fatigue accumulates and becomes chronic, and attending to health and wellness, adjusting expectations of themselves, and preventing burnout become priorities. Questions emerge about how long the event will last and when everything will go back to normal. Family also begins to question how long it will be before they have their loved ones back.

### Reentry

*“I am scared to think about what’s going to happen when everything gets back to normal. I’ve changed too much!” (personal communication).*

Stresses related to transitioning back to normalcy rarely get enough attention. This phase comes with its own unique challenges that, if not addressed, can have a long-term impact on health and relationships at work and home (see more below).

## SOME STRESSORS ARE WORSE THAN OTHERS: “DON’T CALL US HEROES”

It is important to keep in mind that the training and work experience have prepared first responders to manage routine job-related stresses and have allowed them to become aware of the limits of their endurance.

In CEs, the stresses can be extreme, and managing them remains a challenge. Overwhelming stresses make it more difficult to complete professional tasks and increase the probability of making mistakes. Even when responders rest, in their minds, they often still continue working and do not give themselves permission to de-stress.

In addition, some stressors carry a disproportional impact that leaves responders emotionally drained and physically exhausted. During CEs, most stresses come from the long work hours and not having enough time to recuperate, but they also come from uncertainty about the future, a sense of helplessness, and questioning the effectiveness of the work. Surprisingly, several responders told me that being called a hero made them uncomfortable because they believed that they were doing a job where they had to navigate moral dilemmas and make difficult decisions. In this context, being called a hero was experienced as a sort of dubious praise that only added additional pressure and expectations.

Imai et al.^8^ identified factors that influence health worker behavior during epidemics: fear of contagion, concern for family health, interpersonal isolation, quarantine, lack of trust in and support from their organization, information about risks and what is expected of them, and the stigma.

The most toxic stressor named by the trainees was *“betrayal”*—the belief that the organization, community, or family failed them when they needed their support the most. This feeling comes with the sense that the value of work has been minimized, discredited, or not acknowledged. Reduction in salary or inconsiderate words spoken by a superior or a community leader exponentially increase the potential for burnout.

The Substance Abuse and Mental Health Services Administration^9^ has emphasized the importance of restoring predictability, control, and safety in coping with trauma.

During CEs, *predictability* is undermined by the rapidly changing and sometimes chaotic situation. Living in a “one day at a time” mode offers an alternative to long-term planning by focusing on what one does have control over.^2^

In contrast to an unpredictable external reality, establishing routine in one’s personal life strengthens the sense of being *in control*. This can include small daily rituals before going to work and returning home: taking a few minutes for grounding or stretching to tune in. Taking time before going home helps to establish distance between work and personal life. This time allows the worker to decompress before seeing family members who expect a certain emotional presence from their loved ones.

*Safety* of responders is always the biggest concern in CEs. Although it is impossible to guarantee safety during an epidemic, diligently following safety precautions can increase the sense of control over the situation.^9,10^

When not managed, chronic workplace stress can lead to the occupational phenomenon of burnout, which can significantly undermine professional and personal functioning, well-being, and health. The World Health Organization^11^ has identified the universal signs of burnout, such as feelings of energy depletion or exhaustion, increased mental distance from one’s job or feelings of negativism or cynicism toward one’s job, and reduced professional efficacy.

## PEER SUPPORT

Peer support is a critical component of managing psychological needs during a CE. Peer camaraderie and fellowship are all parts of the emergency response culture; first responders often believe that only their peers can fully understand them.

### Peer Support

The objectives of providing peer-to-peer support is to make peers feel noticed, understood, supported, and validated. The following are some brief Dos and Don’ts—please do bear in mind that these are not intended to substitute for professional mental health supports.

Dos

- Check in on each other and be aware of those who are in distress.
- Random acts of kindness, small compliments, and favors can go a long way, eg, offering a bottle of water or box of tissues.
- Be available when someone wants to talk. Be clear with setting time limits. Do not send mixed signals.
- Accept that one can remain distressed even after talking to peers.
- Respect the individual’s way of dealing with difficulties.
- Give feedback if stress affects the job performance or makes the provider unsafe.

Don’ts

- Don’t assume the role of psychotherapist.
- Avoid emotional incongruency, like being Pollyannaish.
- Don’t try to fix others or solve their life problems.
- Don’t suggest they relax.
- Don’t suggest that talking about feelings is going to make them feel better.
- Don’t tell a peer not to think about work at home or about home at work. They may wish they could separate so easily!
- Don’t neglect practical assistance when possible: offering to help with small tasks can go a long way.
- Avoid labeling peers’ behavior or feelings as pathological.
- Don’t forget that you also need supports.

### Crisis Support

Sometimes managers have to provide a brief crisis intervention for an employee who is overwhelmed, develops an emotional breakdown, and struggles to continue working. The goal of crisis support is to assist them with returning to their previous level of functioning, meaning that they continue to treat patients.

Usually, crisis intervention works best when administered by trained external specialists to avoid boundary confusion in work relations. But in its reduced form, a leader (ie, manager or supervisor) can offers immediate emotional supports. Such reduced format of crisis intervention includes components of Psychological First Aid (PFA)^12^ and the ABC model of brief crisis support,^13^ and incorporates structured active listening, which generally has 3 steps: cognitive grounding, emotional support and empathy, and linking to resources. Sample questions to ask to guide this process include: “Tell me what happened”; “What bothers you the most?”; “What helps you to cope?”; and “How can I help you right now?”

A manager can further inquire about resources and supports available for the provider and encourages planning for the self-care. An assessment is being done to determine whether there is a need to recommend higher levels of professional supports, such as the Employee Assistance Program.

### When to Seek Professional Supports?

Additional professional supports are required when individuals are unable to function or sleep, relationships at home or work suffer, and they report suicidal ideations.

## ANGER IS EVERYWHERE AND IT MASKS OTHER FEELINGS

Anger is probably the most common feeling reported during CEs; it is everywhere. Anger management becomes a priority, because when bottled up, suppressed, and unresolved, anger can escalate and be expressed in unsafe ways. It also can affect health. There is nothing cathartic about expressing anger, wrote Stokes,^14^ citing a Mistofsky study that linked angry outbursts to heart attacks and challenged the myth that expressing anger can be cathartic.

Based on my experience, anger often masks and substitutes other feelings that are far more difficult to acknowledge and express, such as fear, helplessness, sadness, and grief. Recognizing these underlying true feelings and expressing them for what they are can help to manage anger.

In addition, in a CE, the responders’ anger (that is often a façade covering helplessness) is sometimes directed at their institutions and the managers thereof, even though they, perhaps imperfectly, are part of the solution. This puts additional pressure on managers.

Providers can also become a target of the patients’ or their families’ misdirected anger, which can be emotionally difficult for the provider who believes that they have been doing everything they could. Here are some tips that can be helpful for managing anger in the workplace during CE:

- Identify the underlying feeling, eg, grief, fear, or sadness, and help to express it directly.
- Respond to feelings, not words.
- When anger is expressed at you, it does not mean it belongs to you, and you do not have to own it.
- Redirect if it gets out of control.
- Avoid placing yourself between a person and their anger, meaning that, if an individual is angry at the agency or someone else, reminding them that you are here in an attempt to help may defuse the situation.
- Chose words that sound both reassuring and grounding. For example, “I promise you that your loved one will get the best care possible.”
- Be mindful of your personal safety and the safety of others. It is important that a person can vent grievances in a way that is not harmful to themselves or others.

## RECOGNIZING AND ADDRESSING FANTASIES

The mental state of extreme concentration in helping professions makes health providers prone to developing illusions and misperceptions. These cognitive aberrations that, in a predictable way, affect how an individual perceives oneself in relation to others are called *fantasies.* Acting out these fantasies threatens the integrity of care, carries the potential for boundary violation, and can lead to burnout; they also affect patient care and the safety of responders.^2^

- The Rescuer fantasy
- The Superman complex
- The Helper’s fantasy.

The *Rescuer fantasy* is a subconscious belief that one must rescue or save another person from something bad, posited as a motive for certain actions or choices (Oxford Dictionary, s.v. “rescuer fantasy”). When acting upon this fantasy, a provider may feel that they alone are charged with the rescue mission and saving human lives.

Similarly, the *Superman fantasy* is based on the belief that Superman is the only one who can solve the problem. In reality, the “superman’s” interventions are not always wanted, and the superman does not take into consideration the natural path of coping with a situation: they offer quick fixes without taking responsibility for finding long-term solutions. It is not uncommon for a superman to actively seek out troubles to solve and thus “save the day.” Overestimating an individual provider’s role undermines collaborative efforts and is detrimental to the team’s work.

Both the Rescuer and the Superman fantasies come with the illusion of omnipotence, irreplaceability, and an inflated perception of one’s own role. They also are associated with perceiving other people as incapable of making decisions.

The *Helper’s fantasy* involves expecting appreciation and gratitude for one’s work. Finding meaning in their work through receiving appreciation makes providers vulnerable and dependent on external approval. Instead of approval, during an epidemic response, helpers may become targets of anger and hostility from those whom they are trying to help.^15^ This age-old phenomenon is reflected in the saying “No good deed goes unpunished!” Although the anger toward helpers is reflective of the whole larger situation, it can take them by surprise and leave them feeling disappointed and hurt. The ground assumption behind this fantasy is challenged by the realization that altruism and acts of kindness increase self-worth, and we do this work for our own self-satisfaction, to feel better about ourselves and fulfil our personal mission—in short, because it feels like the right thing to do.

## SELF-AWARENESS OFFERS CHOICES

To counteract the detrimental impact of emotional over-engagement (common among helping professions in CEs), the *self-awareness* framework emphasizes the awareness of one’s own fantasies, countertransference, and biases, as well as existing choices in the areas of decision-making, risk assessment, and self-care.^2^ Introduction of this concept offers a different perspective on the self-care module, which was decreed an essential component of modern health-care systems governed by regulations and statutes.^16^ The self-care module has become the gold standard for helping professions, and includes recommendations about taking time for oneself through meditation, mindfulness, and grounding exercises. It has shown effectiveness in burnout prevention and has been credited with encouraging helpers to pay attention to their own personal needs.

However, it came as no surprise, that at least half of the trainees who spoke out, have previously pushed back at the offered self-care supports. Self-care, both as a philosophy and a tool, was perceived as a diversion from focusing on patients’ needs, and, most of all, it left the responders feeling like they were not trusted to make decisions about when and how to attend to their own needs. Other named concerns were:

- In the heat of the response, self-care may not be seen as a priority.
- Prioritizing self-care can be negatively perceived by the others, affect team dynamics, and put others at a disadvantage.
- Meditation, grounding, and mindfulness can make it difficult to remobilize and refocus on the work: “It makes me more vulnerable and weaker.”

Instead, in CEs, self-awareness becomes a particularly critical tool for ensuring quality and integrity of care as well as the health and safety of the responders. The concept of self-awareness focuses on realizing and exercising choices that empower responders to reestablish control over their own life and decision-making. The awareness of one’s limits and needs empowers one to make decisions about safety and self-care that are most appropriate for the situation and the professional role.^1^

## SUICIDE RISK AMONG FRONTLINE WORKERS IS REAL!

At times, frontline workers may have doubts about whether they make a difference and can question the meaning of their work. Updegraff et al.^17^ cautioned that premature meaning-making may predict greater posttraumatic stress symptoms. In addition, things appear differently from a distance, and the meaning of the experience most likely will change as time passes.

The exposure to massive suffering and mortality may change the meaning and perceived value of life, resulting in recklessness, taking unnecessary risks, and even suicidal ideations or attempts. This trend has been confirmed by reports about increased suicides among COVID-19 health workers.^18,19^

In humanitarian work, the safety imperative (or absolute priority of safety) refers to the fundamental premise of how we can assess risks and prioritize safety for both the beneficiaries and the providers.^9^ When existential despair and survivor’s guilt sink in, it is very easy to lose perspective and believe that joining the victims is the only moral choice left.^2^ Often in such situations, self-awareness is limited. In my global work, I have used 2 antitheses that serve as a grounding and reminder about the importance of safety:

- *“There is no need to multiply the victims.”* This statement, which is widely repeated among first responders, suggests that self-sacrifice is not going to help resolve the situation and means that fewer patients will be helped.
- *“Your life doesn’t belong to you only.”* Questioning the value and meaning of life is often driven by identification with the victims; this statement reminds responders about their responsibility to their family, who worries about them and expects that their loved one will do everything they can to stay safe, if not for themselves then for the family’s sake.

## IT IS EASIER TO ADAPT TO EMERGENCY THAN TO REENTRY

Various studies of emergency responders have demonstrated that reentry can be psychologically challenging and has been associated with increased risks for substance abuse, depression, family breakup, and suicide.^20^ Both aid workers and frontline health-care responders have disclosed their fears about what would happen after the emergency was over. And indeed, some have reported easier psychological adjustment to the emergency mode than readjustment to “normal life.”^2,21^

To prepare and plan for reentry, emergency workers need to be aware of these challenges and know how and when to reach out for professional help. When work-life starts returning to normalcy, providers can anticipate dealing with following issues:

- Not being fully emotionally present with family and friends
- Not being able to experience happiness and other strong emotions
- A possible increase in conflicts at home and work
- Anhedonia (lack of energy and drive)
- Poor sleep, anxiety, depression, irritability, and anger
- Social isolation, having no desire or energy to socialize
- Traumatic symptoms, such as intrusions, flashbacks, nightmares, or survivor guilt
- Increased alcohol and drug use
- Being “stuck in the past,” thinking and talking only about the CE
- Worsening somatic problems, such as hypertension and diabetes

Usually, these symptoms go away fairly quickly. If they do not improve with time, get worse, or lead to developing the feeling that life is not worth living or suicidal thoughts, it is important to seek professional supports.

### How to Prepare for Reentry

- Prepare your family: Explain to your family ahead of time that you may be emotionally distant for some time and that it does not mean that you love them any less.
- Prepare yourself: Be ready for a period of “sluggishness” and physical and mental fatigue. Making too many plans may turn out to be impossible to achieve and can result in further disappointment in oneself. Allow yourself to be tired, sad, and less productive.
- Prepare your social network and community: As the response to Ebola demonstrated, the stigma of the disease can extend to the health-care providers by virtue of their work with the infected patients.^22^ It is important to educate the community about the nature of your work (rumors are always worse) and explain that you are not contagious. Because of the associated stigma, sometimes it makes sense to avoid visiting children’s playgrounds and large public gatherings for some time.

## CONCLUSIONS

The response to CEs has elevated the role of health-care and disaster management organizations in supporting the workforce and has provided opportunities for transformative change. Agencies that actively work on adjusting program policies and procedures are likely to have more success in providing care, retaining clients, reducing staff turnover, and achieving positive outcomes.^9^

### Recommendations for Organizations and Managers

- Acknowledge the work of frontline health-care workers. A lack of recognition from superiors impacts staff morale and can lead to loss of trust in the organization, burnout, and staff turnover.
- Strengthen organizational support for managers. Managers have to mediate the pressure coming from both the organization and the employees; they often become the target of the anger and expressed frustration stemming from the overall situation.
- Establish an external referral system where responders can receive professional mental health support. Co-located mental health units may not be equipped to provide crisis response and psychological supports to employees in an emergency.
- Trust is essential. Creating spaces of safety where workers can come together and openly share their feelings without fear of repercussions can help with establishing the workplace as a place of safety.
- Managers need to have a basic training in self-awareness, psychological first aid, and basic crisis support. This includes recognizing and managing fantasies, excessive risk-taking, and suicidal behavior.
